# Genetics and Vitamin D Interactions in Osteoporosis: A Path to Precision Medicine

**DOI:** 10.1111/jcmm.70780

**Published:** 2025-08-19

**Authors:** Sepideh Abdollahi, Forough Taheri, Amirhossein Sangi Nasab Lahijan, Saba Hatefi Shoga, Ali Didehban, Saeid Doaei

**Affiliations:** ^1^ Department of Medical Genetics, School of Medicine Tehran University of Medical Sciences Tehran Iran; ^2^ Metabolic Disorders Research Centre, Endocrinology and Metabolism Molecular‐Cellular Sciences Institute Tehran University of Medical Sciences Tehran Iran; ^3^ Department of Genetics, Faculty of Basic Sciences Shahrekord University Shahrekord Iran; ^4^ Human Genetics Research Center Baqiyatallah University of Medical Sciences Tehran Iran; ^5^ Department of Community Nutrition, Faculty of Nutrition Sciences and Food Technology, National Nutrition and Food Technology Research Institute Shahid Beheshti University of Medical Sciences, Tehran Tehran Iran; ^6^ Unit of Nutrition and Cancer, Cancer Epidemiology Research Program, Catalan Institute of Oncology Bellvitge Biomedical Research Institute (IDIBELL), L'Hospitalet de Llobregat Barcelona, Spain. Barcelona Spain

**Keywords:** bone mineral density (BMD), genetic polymorphisms, osteoporosis, precision medicine, vitamin D metabolism

## Abstract

Osteoporosis is a systemic skeletal disease characterized by reduced bone mineral density (BMD) and increased fracture risk; it poses a significant global health challenge. The multifactorial pathogenesis of osteoporosis involves complex interactions between genetic factors and vitamin D metabolism, particularly involving key genes such as the vitamin D receptor (VDR), CYP27B1 and CYP24A1. Polymorphisms in these genes, including FokI, BsmI, TaqI and ApaI in the VDR gene, have been associated with variations in BMD, fracture susceptibility and differential responses to vitamin D supplementation, underscoring the importance of personalized medicine. Genome‐wide association studies (GWAS) have identified over 500 loci, including WNT16, ESR1 and SOST, linked to osteoporosis‐related traits, underlining the disease's polygenic nature and the impact of gene–environment interactions, including dietary vitamin D intake, sun exposure and gene variations. Despite these advancements, translating genetic insights into clinical practice remains challenging, especially due to the variability in genetic determinants and limited access to genotype assessment such as gene sequencing. This review advocates for precision medicine approaches to osteoporosis management. By addressing the gaps in the studies on osteoporosis aetiology, integrating genetic screening into routine diagnosis and care and promoting collaborative efforts in genomics, nutrition and public health, the global burden of osteoporosis can be significantly reduced. This approach offers a promising pathway to improve patient outcomes and advance personalized medicine strategies for osteoporosis as a debilitating condition.

## 1 Introduction

Osteoporosis is a chronic skeletal disease marked by reduced bone mineral density (BMD) and increased fracture risk [1]. Its prevalence rises with age, particularly in postmenopausal women due to oestrogen decline, which accelerates bone loss [2]. Beyond hormonal and structural factors, growing evidence highlights the role of gene–nutrient interactions—particularly between vitamin D metabolism and genetic polymorphisms—as critical contributors to osteoporosis risk and variability in treatment response. The global prevalence of osteoporosis imposes considerable healthcare costs and societal burdens, with annual osteoporosis‐related fracture expenses in the United States alone estimated at $17.9 billion [3]. Beyond economic impacts, osteoporosis is associated with reduced quality of life, long‐term disability and increased mortality following fractures, emphasising its public health significance [4]. Risk factors for osteoporosis include non‐modifiable factors like genetics and modifiable elements, such as diet—particularly vitamin D deficiency. The interplay between gene‐diet interactions plays a crucial role in osteoporosis pathogenesis [5].

Vitamin D is essential for bone health, primarily through its role in regulating calcium and phosphate metabolism, which are critical for bone mineralization. Vitamin D deficiency leads to increased bone resorption, decreased BMD and heightened susceptibility to osteoporosis. Its active form, 1,25‐dihydroxyvitamin D, is crucial for promoting osteoblast function and osteoclast activity, supporting bone remodelling. Vitamin D supplementation is a common therapeutic strategy to address deficiencies and mitigate osteoporosis risk [6].

While vitamin D plays an essential role in bone metabolism, clinical trials have yielded inconsistent results regarding its supplementation's efficacy in reducing fracture incidence [7]. Some studies have found no significant reduction in fractures despite correcting vitamin D deficiency, suggesting that vitamin D alone may not be sufficient for fracture prevention and that other factors—including calcium intake, physical activity and comorbidities—must be considered [8]. Vitamin D status is influenced by both genetic and environmental factors. Most of the biological actions of vitamin D are mediated by the vitamin D receptor (VDR), which binds to 1,25‐dihydroxyvitamin D3 to regulate skeletal development, maintain bone architecture and influence hormone secretion and immune function [9]. The VDR gene has been identified as a key regulator of bone strength and metabolism, with its polymorphisms significantly implicated in osteoporosis risk [10]. In fact, genetic factors are estimated to account for 50%–85% of the variance in bone mineral density (BMD), underscoring the importance of investigating genetic contributions to osteoporosis pathogenesis [11].

Observational studies have reported associations between single‐nucleotide polymorphisms (SNPs) in vitamin D‐related genes and circulating vitamin D levels [12]. However, the influence of these SNPs on individual responses to dietary vitamin D and vitamin D supplementation remains underexplored. Understanding such genetic variability is essential for the development of personalized medicine strategies, enabling more precise vitamin D supplementation and osteoporosis prevention tailored to individual genetic profiles. Recent advancements in functional genomics have paved the way for interventional studies that assess individual‐level variability in response to supplementation, highlighting the potential for personalized approaches to osteoporosis management. Hence, the primary aim of this paper is to provide a comprehensive review of the interaction between genetics and vitamin D in the context of osteoporosis, focusing on how vitamin D affects osteoporosis‐related genes and how polymorphisms influence the efficacy of vitamin D supplementation.

## 2 Vitamin D and Bone Health

### 2.1 Vitamin D Synthesis and Activation

Vitamin D metabolism is a highly regulated process essential for its activation and function as a hormone. Following synthesis in the skin from 7‐dehydrocholesterol under ultraviolet B radiation or through dietary intake, vitamin D undergoes a two‐step enzymatic hydroxylation [13]. The first step occurs in the liver, where cytochrome P450 enzyme CYP2R1 converts vitamin D to 25‐hydroxyvitamin D (25(OH)D), the predominant circulating form and a critical biomarker of vitamin D status. The second step occurs in the kidneys, where the enzyme CYP27B1 hydroxylates 25(OH)D to produce the biologically active form, 1,25‐dihydroxyvitamin D (1,25(OH)2D). This active metabolite, calcitriol, has crucial physiological roles such as calcium and phosphate homeostasis [14], which are essential for bone health and various metabolic functions.

### 2.2 Binding to VDR and Regulation of Calcium

Calcitriol exerts its effects by binding to the vitamin D receptor (VDR), a nuclear receptor expressed in target tissues such as the intestines, kidneys and bones. In the intestines, this binding enhances the transcription of genes encoding calcium‐binding proteins, thereby increasing the efficiency of dietary calcium absorption. In the kidneys, calcitriol facilitates the reabsorption of calcium and phosphate, thereby reducing excretion and conserving these essential minerals [15].

### 2.3 Effects on Bone Remodelling: Osteoblasts and Osteoclasts

Additionally, calcitriol influences bone remodelling by promoting the differentiation and activity of osteoclasts, cells responsible for bone resorption, which releases calcium and phosphate into the bloodstream [16]. Regarding the effects of 1,25‐dihydroxyvitamin D on bone remodelling, it directly influences the activity of osteoblasts, the bone‐forming cells. These cells express the vitamin D receptor (VDR), which binds to 1,25(OH)_2_D and regulates the expression of genes critical for osteoblast differentiation and mineralization, such as alkaline phosphatase, osteocalcin and osteopontin. In addition to its direct effects, 1,25(OH)_2_D indirectly stimulates osteoclast activity by inducing osteoblasts to express receptor activator of nuclear factor kappa‐Β ligand (RANKL). RANKL binds to its receptor RANK on osteoclast precursors, promoting their differentiation and activation. Furthermore, 1,25(OH)_2_D exerts direct effects on osteoclasts, as both mature osteoclasts and their precursors express VDR. This interaction enhances osteoclastogenesis by upregulating transcription factors like NFATc1, which plays a key role in osteoclast differentiation and activity. These actions together ensure tight regulation of bone remodelling. Vitamin D plays a central role in this process [17, 18].

**FIGURE 1:**
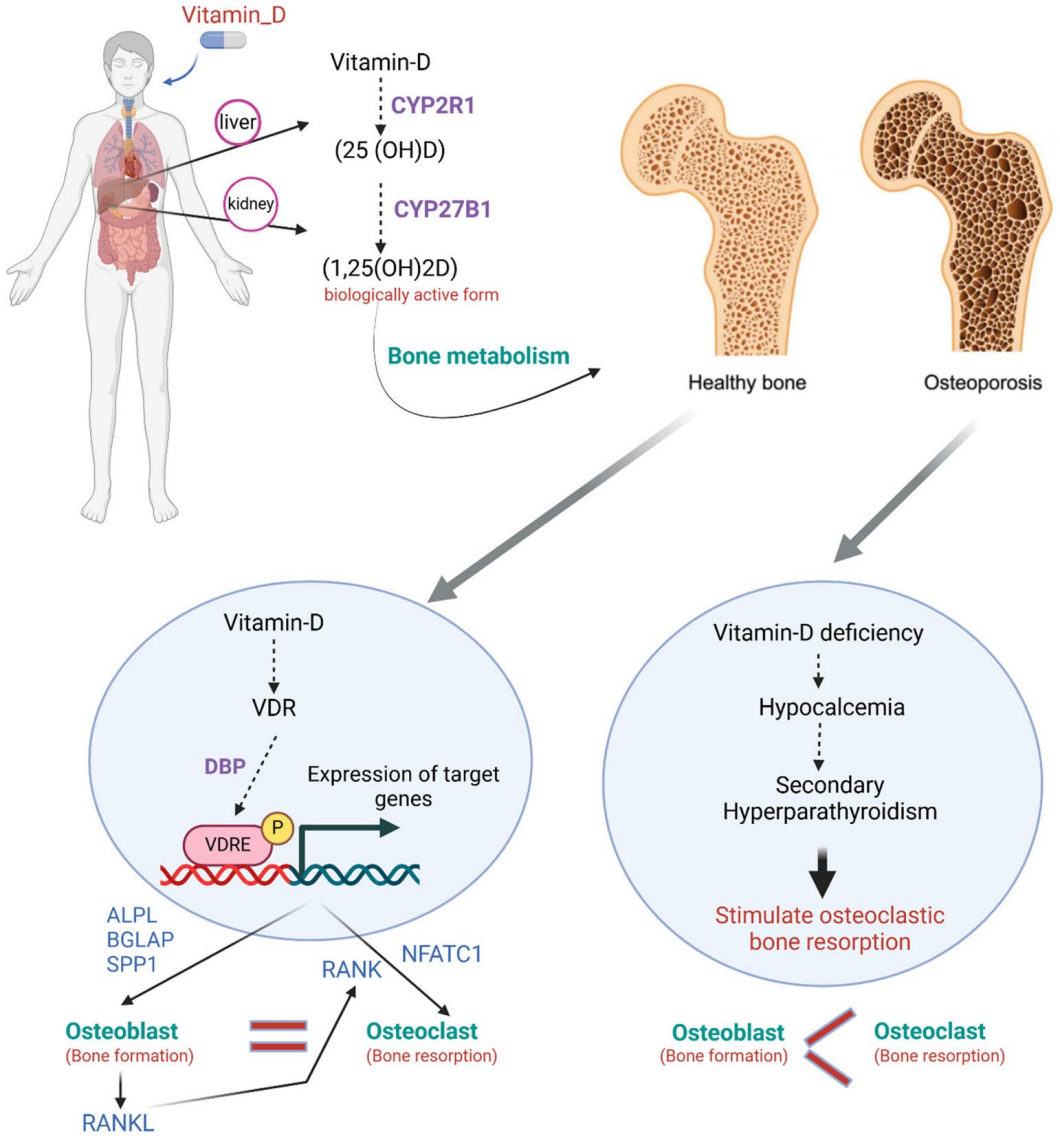
Vitamin‐D metabolism and its impact on bone health. (1,25(OH)2D), 1,25‐dihydroxyvitamin D; (25(OH)D), 25‐hydroxy‐cholecalciferol; ALPL, Alkaline phosphatase; BGLAP, Bone gamma‐carboxyglutamate protein; CYP27B1, Cytochrome P450 Family 27 Subfamily B Member 1; CYP2R1, Cytochrome P450 Family 2 Subfamily R Member 1; DBP, vitamin D‐binding protein; NFATC1, Nuclear factor of activated T‐cells; RANK, Receptor activator of nuclear factor kappa‐Β; RANKL, Receptor activator of nuclear factor kappa‐Β ligand; SPP1, Osteopontin; VDR, Vitamin‐D receptor; VDRE, Vitamin‐D response element.

Thus, the balanced regulation of osteoblast and osteoclast activities by 1,25(OH)_2_D is vital for maintaining bone homeostasis. Disruptions in vitamin D signalling can lead to skeletal disorders characterized by impaired bone remodelling, such as osteoporosis and rickets [17]. Figure 1 illustrates the metabolic pathway of vitamin D, including its synthesis, activation and the mechanisms by which 1,25‐dihydroxyvitamin D influences bone remodelling through effects on osteoblasts and osteoclasts. This schematic highlights how disruptions at any stage—from synthesis to receptor binding—can impair skeletal health, emphasising the relevance of vitamin D signalling to osteoporosis pathogenesis and the need for genetically informed treatment strategies. Importantly, the magnitude and direction of these effects are not uniform across individuals. Genetic polymorphisms in key regulators of vitamin D metabolism and signalling—such as the VDR gene (e.g., FokI, TaqI), CYP27B1 and GC—can modulate the efficacy of 1,25(OH)_2_D‐mediated pathways. These variations influence receptor activity, hormone conversion efficiency and vitamin D bioavailability, ultimately affecting how bone cells respond to vitamin D at the molecular level [19].

## 3 Vitamin D Deficiency in Osteoporosis

### 3.1 Prevalence and Risk Factors

Vitamin D deficiency is a prevalent global health concern, affecting diverse populations across various regions (Table 1). The definition of deficiency varies, with thresholds of serum 25‐hydroxyvitamin D [25(OH)D] levels ranging from less than 25 nmol/L to 50 nmol/L, significantly influencing prevalence estimates. According to current clinical guidelines, the Endocrine Society defines vitamin D deficiency as serum 25(OH)D levels below 50 nmol/L (20 ng/mL), insufficiency as 52.5–72.5 nmol/L (21–29 ng/mL) and sufficiency as levels of 75 nmol/L (30 ng/mL) or higher [20]. In contrast, the Institute of Medicine (IOM) considers levels below 30 nmol/L (12 ng/mL) as deficient and recommends levels above 50 nmol/L (20 ng/mL) as sufficient for most of the population [21]. Even with conservative thresholds (e.g., < 25/30 nmol/L), substantial deficiency rates are observed in both low‐ and high‐income countries. The prevalence of vitamin D deficiency is significantly higher among the elderly, obese individuals, nursing home residents and hospitalized patients. For example, vitamin D status tends to decline with age due to reduced dermal synthesis and limited outdoor activity, with 61% of elderly individuals in the United States classified as deficient. Among infants, limited sun exposure and exclusive breastfeeding without supplementation are major risk factors. In the United States, 47% of African American infants and 56% of Caucasian infants are vitamin D deficient, while over 90% of infants in countries such as Iran, Turkey and India are affected. In adults, approximately 35% of the US adult population is vitamin D deficient, compared to over 80% in Pakistan, India and Bangladesh, which highlights disparities related to geographic and lifestyle factors [22, 23]. Obese individuals, regardless of latitude or age, show a 35% higher prevalence of deficiency, likely due to sequestration of vitamin D in adipose tissue [24]. Individuals with darker skin tones or those who use extensive clothing that covers most of the skin, especially in Middle Eastern countries, are also at increased risk [25, 26]. The primary cause of vitamin D deficiency is inadequate cutaneous synthesis due to limited exposure to ultraviolet B (UVB) radiation. Factors contributing to this include high latitudes, seasonal variations, air pollution and cultural practices that limit sun exposure. Personal characteristics such as older age, higher body mass index and darker skin pigmentation also impede dermal vitamin D synthesis [27]. Dietary intake of vitamin D is typically low, as few foods naturally contain significant amounts of the vitamin. This insufficiency is exacerbated by limited consumption of vitamin D‐rich foods and low rates of supplementation. Consequently, addressing vitamin D deficiency through dietary means poses challenges, necessitating strategies like food fortification and supplementation to improve population vitamin D status [28].

**TABLE 1:** Summary of global vitamin D deficiency prevalence across population groups.

Population group	Region/country	Prevalence of vitamin D deficiency (%)
Infants	USA—African American	47
Infants	USA—African American	56
Infants	Iran, Turkey, India	> 90
Adults	USA	35
Adults	Pakistan, India, Bangladesh	> 80
Elderly	USA	61
Elderly	Turkey	90
Elderly	India	96
Elderly	Pakistan	72
Elderly	Iran	67

### 3.2 Physiological Impact of Deficiency

Vitamin D deficiency has profound implications for bone health, primarily through its role in calcium and phosphate homeostasis. Insufficient levels of vitamin D lead to decreased intestinal absorption of calcium, resulting in hypocalcaemia [29]. This condition triggers secondary hyperparathyroidism, characterized by elevated parathyroid hormone (PTH) levels, which in turn stimulate osteoclastic bone resorption to maintain serum calcium concentrations [30]. The increased bone resorption surpasses bone formation, leading to a net loss of bone mineral density (BMD). Consequently, vitamin D deficiency is related to a higher risk for osteomalacia in adults and rickets in children, conditions marked by impaired bone mineralization. Moreover, the reduction in BMD induced by vitamin D deficiency elevates the risk of osteoporosis and associated fractures [31].

### 3.3 Evidence From Epidemiological and Interventional Studies

Epidemiological studies have demonstrated that the prevalence of vitamin D deficiency is significantly higher among osteoporotic patients, with studies indicating that over 50% of postmenopausal women treated for osteoporosis have insufficient vitamin D levels (< 30 ng/mL). Interventional studies demonstrate that supplementation with 800–1000 IU of vitamin D daily, combined with calcium, reduces fracture risk and improves bone mineral density [32]. In addition, serum 25‐hydroxyvitamin D (25(OH)D) levels below 12 ng/mL were reported to be associated with a 3.1‐fold increased risk of fractures in individuals aged 65–75 years, even after adjusting for confounding factors such as age, sex, season of blood sampling and physical performance. This study, conducted among 1311 community‐dwelling older adults, highlighted that 17.5% of participants had serum 25(OH)D levels below the critical threshold of 12 ng/mL. Interestingly, the relationship between vitamin D deficiency and fracture risk was more evident in younger seniors (65–75 years), with diminished significance in those aged 75 years or older, potentially due to multifactorial causes of fractures in the older population. The study underscores the importance of maintaining adequate vitamin D levels, suggesting that serum concentrations above 30 ng/mL were associated with the lowest risk of fractures [33]. Moreover, the association of vitamin D deficiency with an increased risk of osteoporosis and related complications such as fractures is emphasized in the National Osteoporosis Society guidelines. The guidelines highlight that patients with musculoskeletal symptoms or those undergoing osteoporosis treatment should have their vitamin D levels measured and corrected if necessary. Interventional strategies include the use of oral vitamin D3 supplementation, with loading doses of approximately 300,000 IU administered over 6–10 weeks for rapid correction, followed by maintenance therapy of 800–2000 IU daily [34] (Figure 1).

Hence, understanding the prevalence, causes and clinical impact of vitamin D deficiency is essential for effective public health and clinical strategies. Acknowledging these factors allows for better management of osteoporosis risk, especially in high‐risk populations.

## 4 Genetic Factors in Osteoporosis

### 4.1 Heritability and Polygenic Nature

Osteoporosis, characterized by reduced bone mineral density (BMD) and increased fracture susceptibility, is a highly heritable disease with genetic factors accounting for approximately 50%–85% of BMD variation [35, 36]. Twin and family‐based studies have consistently demonstrated a strong heritable component to osteoporosis‐related traits, including peak bone mass, skeletal geometry and bone turnover [35, 37]. In contrast, fracture risk is influenced by a wider range of factors—including fall risk, muscle strength, medication use and environmental hazards—which dilute the relative contribution of genetics. Therefore, while BMD is a quantitative trait directly regulated by genetic pathways affecting bone formation and mineralization, fractures are the clinical outcome of multiple interacting systems, making their heritability substantially lower. Fracture risk, though influenced by both genetic and environmental factors, is moderately heritable, with estimates decreasing significantly with age [35].

### 4.2 Gene Contributions to Bone Biology

Genetic predisposition to osteoporosis arises from the interplay of multiple genes, each contributing small effects, making osteoporosis a polygenic disorder [36]. Monogenic forms of bone diseases, such as osteogenesis imperfecta and sclerosteosis, have highlighted the involvement of key genes like COL1A1, COL1A2, SOST and LRP5. COL1A1 and COL1A2 encode type I collagen, a major structural protein in bone; mutations in these genes disrupt collagen formation and lead to brittle bones. SOST encodes sclerostin, a protein that inhibits bone formation by antagonising the Wnt signalling pathway, while LRP5 is a co‐receptor in the same pathway, promoting osteoblast activity and bone accrual [36, 38]. Lifestyle factors, including physical activity and calcium intake, interact with this genetic framework to influence the disease phenotype [35, 39].

### 4.3 Discoveries From GWAS

Genome‐wide association studies (GWAS) have significantly advanced our understanding of osteoporosis genetics, identifying hundreds of loci associated with BMD, osteoporosis and fracture risk. Notably, loci such as WNT16, ESR1, SOST and RANKL have been consistently replicated and are implicated in critical pathways of bone biology, including the Wnt signalling pathway and osteoclastogenesis [40, 41, 42]. The largest GWAS meta‐analyses involving populations of European and Asian descent have discovered novel loci like FAM210A, GRB10 and ETS2, which broaden the biological understanding of bone strength and fragility. FAM210A is thought to regulate mitochondrial function and osteoblast differentiation, GRB10 modulates insulin and IGF‐1 signalling that influence bone mass and ETS2 is a transcription factor involved in cell proliferation and differentiation processes, potentially impacting bone turnover [36, 41]. Furthermore, GWAS findings have identified loci affecting not only BMD but also bone geometry, cortical thickness and trabecular microarchitecture [36]. Despite these discoveries, the challenge of ‘missing heritability’ persists, where identified genetic variants explain only a fraction of the heritable variance in osteoporosis, suggesting the need for larger multiethnic studies and functional validation of loci.

### 4.4 Role of SNPs and Polygenic Scores

Single‐nucleotide polymorphisms (SNPs) serve as critical genetic markers for understanding osteoporosis risk and have elucidated the polygenic nature of the disease. The identified SNPs through GWAS, such as rs1021188 near RANKL and rs597319 near TMEM135, have shown significant associations with both BMD and fracture risk [40, 41]. SNPs within genes like WNT16 and ESR1 highlight the importance of the Wnt and oestrogen signalling pathways in bone remodelling and mineralization [41, 42]. However, the effect sizes of individual SNPs are generally small, necessitating the integration of multiple SNPs into polygenic risk scores (PRS) for improved fracture risk prediction [37, 41]. PRS are quantitative measures that aggregate the effects of SNPs across the genome to estimate an individual's genetic predisposition to a specific trait or disease.

Additionally, SNPs often reside in non‐coding regions and influence gene expression through epigenetic mechanisms, further emphasising the need for functional studies to understand their biological roles [42].

### 4.5 Importance of Ethnic Diversity

Importantly, population‐specific SNP variations underscore the importance of conducting GWAS in diverse ethnic groups to ensure broader applicability of findings in clinical settings [38, 40]. These findings suggest that genetic and epigenetic variations influence the risk of developing osteoporosis and the response to vitamin D. Further research into these genetic factors may provide a foundation for personalised prevention and treatment strategies based on individual genetic profiles.

## 5 Vitamin D Receptor (VDR) Gene

The vitamin D receptor (VDR) gene encodes a ligand‐dependent nuclear transcription factor that mediates the genomic effects of 1,25‐dihydroxyvitamin D3, the active form of vitamin D. The VDR gene spans approximately 75 kb of DNA and comprises 11 exons, with the structural coding region consisting of eight exons (exons 2–9), while the non‐coding 5′‐end includes three exons (1A, 1B and 1C) involved in alternative splicing [43]. The VDR gene's promoter region is GC‐rich and lacks a canonical TATA box but contains multiple binding sites for transcription factors such as SP1, which are critical for basal transcriptional regulation [43]. The protein produced by the VDR gene consists of several functional domains including an N‐terminal DNA‐binding domain (DBD), a flexible hinge region and a C‐terminal ligand‐binding domain (LBD). The DBD contains two zinc finger motifs that enable VDR to recognize and bind to vitamin D response elements (VDREs) in the promoter regions of target genes. Specifically, residues in the first zinc finger structure, such as P‐box and S‐box, mediate DNA binding, while the second zinc finger contributes to heterodimerization with the retinoid X receptor (RXR) [44, 45]. The ligand‐binding domain (LBD) stabilizes upon 1,25(OH)_2_D_3_ binding, adopting a conserved three‐layered α‐helical sandwich structure. Critical residues, such as Ser237, Arg274 and His305, form hydrogen bonds with the ligand's hydroxyl groups to anchor the molecule within the ligand‐binding pocket (LBP) [45]. Functional activation of the VDR involves its heterodimerization with RXR and recruitment of coregulators, which drive transcriptional activation or repression of target genes, modulating processes such as calcium homeostasis, cellular differentiation and immune response. Mutations in VDR can impair ligand or DNA binding, as seen in hereditary vitamin D‐resistant rickets (HVDRR), which underscores the gene's pivotal role in skeletal development and mineral metabolism [45]. This finely tuned structural organization and function of VDR allow it to regulate diverse cellular processes, providing insights into its critical role in bone health and disease.

Polymorphisms in the VDR gene, including FokI (rs2228570), BsmI (rs1544410) and TaqI (rs731236), have been extensively investigated for their associations with osteoporosis risk and BMD. The VDR gene, located on chromosome 12q13.11, encodes the receptor that mediates the biological effects of vitamin D, a critical regulator of calcium homeostasis and bone metabolism. Polymorphisms such as FokI, BsmI and TaqI are widely studied due to their potential impact on BMD and susceptibility to osteoporosis. The FokI polymorphism, situated in exon 2, alters the start codon and can result in two VDR protein variants differing in their activity. The minor ‘f’ allele is linked to reduced VDR function and has been associated with lower BMD and an increased risk of osteoporosis, particularly in populations of Northwest India [46] and Asian ethnicities [47]. The BsmI polymorphism, which occurs in an intronic region, is thought to affect VDR mRNA stability and expression. Research has shown that the ‘b’ allele of the BsmI polymorphism correlates with decreased BMD and higher osteoporosis risk, particularly in Asian populations, as highlighted in meta‐analyses [48]. The TaqI polymorphism, located in exon 9, influences VDR's biological activity and the presence of the ‘t’ allele has been linked to reduced BMD at key sites such as the lumbar spine and femoral neck. In contrast, studies on Iranian menopausal women from Zanjan province demonstrated no significant relationship between TaqI and ApaI polymorphisms and osteoporosis risk, suggesting the influence of these polymorphisms may vary across ethnic groups [49]. Also, in Saudi Arabian women, a significant association was observed between TaqI (tt genotype) and osteoporosis, with the minor alleles ‘t’ and ‘a’ of ApaI polymorphisms contributing to lower BMD [50]. Meta‐analyses have further reinforced ethnic variability in the association of VDR polymorphisms with osteoporosis. For instance, Caucasian postmenopausal women with the ApaI ‘aa’ genotype were found to have higher BMD in certain regions, whereas in Asian populations, the BsmI and FokI polymorphisms revealed a stronger correlation with decreased BMD and osteoporosis risk [47, 48]. Furthermore, studies on White British men revealed that the TaqI polymorphism significantly influences lumbar spine BMD, with the ‘CC’ genotype being less frequent in controls, thereby suggesting its potential as a genetic risk factor for osteoporosis in males [51]. The discrepancies in findings across studies highlight the multifactorial nature of osteoporosis, involving genetic, environmental and lifestyle factors. Differences in dietary calcium intake, vitamin D levels and ethnic‐specific genetic variations may explain the observed inconsistencies. Populations such as those in the Middle East and Iran are particularly prone to vitamin D deficiency, which, in conjunction with VDR polymorphisms, exacerbates osteoporosis risk [49, 50]. In addition, a meta‐analysis encompassing 67 eligible genetic studies reveals significant ethnic‐specific associations between ApaI (rs7975232), BsmI (rs1544410), FokI (rs10735810) and TaqI (rs731236) polymorphisms and postmenopausal osteoporosis (PMOP) [52]. In Caucasian populations, the ApaI polymorphism demonstrated a significant protective effect in the dominant model (OR = 0.77, *p* = 0.007), while the BsmI polymorphism was associated with reduced osteoporosis risk (OR = 0.69, *p* = 0.002) and a lower frequency of the minor allele ‘b’ in affected individuals. Additionally, the TaqI polymorphism exhibited a significant association under the recessive model, with the ‘tt’ genotype increasing susceptibility to osteoporosis (OR = 1.32, *p* = 0.01). Conversely, in Asian populations, the FokI polymorphism demonstrated a robust association with osteoporosis risk. The dominant model indicated a protective effect of the major ‘F’ allele (OR = 0.61, *p* = 0.0001), while the recessive model revealed a strong association of the ‘ff’ genotype with osteoporosis susceptibility (OR = 2.02, *p* = 0.001). These findings suggest that the FokI polymorphism may play a more prominent role in Asian populations compared to Caucasians, likely due to differences in genetic predisposition, vitamin D metabolism and environmental factors such as diet and sun exposure. It is worth noting that regional studies, including those conducted in Iran, confirm the relevance of VDR gene polymorphisms with susceptibility to PMOP. For example, studies from Iran and surrounding regions highlight the presence of significant genetic variations in ApaI and BsmI polymorphisms but often yield inconsistent results due to small sample sizes, differing methodologies and regional variations in vitamin D deficiency prevalence [52]. Overall, while FokI, BsmI and TaqI polymorphisms show varying degrees of association with osteoporosis risk and BMD, the results remain inconclusive due to genetic heterogeneity and population‐specific influences. Further large‐scale, multiethnic studies are required to confirm these associations and explore gene–environment interactions to better understand the role of VDR polymorphisms in bone health.

Vitamin D, primarily in its active form 1,25‐dihydroxyvitamin D3 (1,25(OH)₂D₃), plays a critical role in regulating bone metabolism through its effects on osteoblasts, osteoclasts and the extracellular matrix. The primary mechanism involves the activation of the VDR, which interacts with Vitamin D Response Elements (VDREs) in target genes, influencing transcriptional activity and promoting osteogenesis [53, 54]. Direct actions of 1,25(OH)_2_D_3_ on osteoblasts include the upregulation of key mineralization‐related genes such as alkaline phosphatase (ALPL), osteocalcin (BGLAP) and osteopontin (SPP1), which contribute to the synthesis and mineralization of the extracellular matrix [53, 55]. Microarray analyses further reveal that 1,25(OH)₂D₃ modulates over 150 genes in human osteoblasts, influencing pathways related to coagulation, immune response and neurotransmitter transporters, demonstrating its broad regulatory impact beyond skeletal systems [55]. The interplay between vitamin D signalling and bone‐related gene networks is multifaceted. For instance, vitamin D stimulates the transcription of RANKL (Receptor Activator of Nuclear Factor κB Ligand), a critical osteoblast‐derived cytokine that promotes osteoclastogenesis, thereby coordinating bone resorption and formation [56]. However, this action is tightly regulated to prevent excessive bone loss, as 1,25(OH)_2_D_3_ also induces the expression of osteoprotegerin (OPG), a decoy receptor that inhibits RANKL activity [54]. Additionally, vitamin D signalling interacts with other pathways, such as the MAPK signalling pathway, where VDR activation upregulates microRNAs like miR‐140‐5p to inhibit osteoblast apoptosis and promote differentiation [57]. This intricate crosstalk between vitamin D signalling and microRNA networks highlights its role in maintaining skeletal homeostasis at the molecular level. Emerging evidence further supports the autocrine regulation of vitamin D metabolism within bone tissue. CYP27B1, the enzyme responsible for converting 25(OH)D to its active form 1,25(OH)_2_D_3_, is expressed in osteoblasts, osteocytes and osteoclasts, suggesting local production of active vitamin D within bone microenvironments [54]. The coordinated expression of CYP27B1 with transcription factors such as RUNX2 and resorption markers like TRAP underscores the dual role of vitamin D in bone formation and remodelling [54, 57]. Importantly, studies suggest that 1,25(OH)₂D₃ may counteract the effects of inflammatory mediators like IFN‐β, promoting mineralization even under pro‐inflammatory conditions, though further research is required to confirm these mechanisms and their clinical relevance [56]. Moreover, dietary calcium and vitamin D intake may modify the effects of VDR genotypes on bone health. For example, individuals carrying certain VDR polymorphisms (e.g., FokI, BsmI) may exhibit different responses to supplementation, with higher intake potentially mitigating genotype‐related risks for low bone mineral density. In summary, vitamin D exerts its effects on bone metabolism through direct modulation of gene expression, regulation of osteoblast function and osteoclast activity and integration with signalling pathways such as MAPK and immune responses. The autocrine control of vitamin D metabolism within bone tissue further enhances its regulatory precision, positioning 1,25(OH)_2_D_3_ as a pivotal factor in skeletal development, maintenance and pathology.

### 5.1 Other Genes Involved in Bone Health

The regulation of calcium homeostasis and bone remodelling is tightly controlled by genes encoding key enzymes such as CYP27B1 and CYP24A1, which are critical in the vitamin D metabolic pathway. CYP27B1 encodes 25‐hydroxyvitamin D‐1α‐hydroxylase, an enzyme responsible for converting 25‐hydroxyvitamin D [25(OH)D] into its active form, 1,25‐dihydroxyvitamin D3 [1,25(OH)_2_D_3_], while CYP24A1 encodes 24‐hydroxylase, which inactivates 1,25(OH)_2_D_3_ by hydroxylating it into 24,25‐dihydroxyvitamin D [58]. These enzymes are critical in maintaining calcium and phosphate balance, directly influencing bone mineralization. Dysregulation of CYP27B1 can lead to reduced levels of active vitamin D, impairing calcium absorption and increasing the risk of bone disorders such as rickets and osteomalacia. Conversely, overexpression of CYP24A1 has been associated with accelerated degradation of 1,25(OH)_2_D_3_, leading to vitamin D insufficiency and subsequent reductions in bone mineral density (BMD) [58]. The interplay between these genes and the Vitamin D Receptor (VDR) further highlights their importance in bone metabolism. VDR activation in osteoblasts initiates the transcription of downstream target genes involved in osteogenesis, including alkaline phosphatase (ALPL) and osteocalcin (BGLAP) [58, 59]. Genetic variations or single‐nucleotide polymorphisms (SNPs) in CYP27B1 and CYP24A1 have been associated with susceptibility to osteoporosis, as they modulate vitamin D availability and calcium signalling. For example, decreased expression or loss‐of‐function mutations in CYP27B1 reduce circulating 1,25(OH)_2_D_3_ levels, impairing calcium absorption and weakening bone mineralization [60]. In contrast, CYP24A1 overactivity may exacerbate bone resorption due to increased vitamin D catabolism, as demonstrated in hypercalciuric stone formers where 1,25(OH)_2_D_3_ levels were elevated relative to CYP24A1 activity [58]. The broader genetic networks influencing bone remodelling extend to pathways like the Wnt/β‐catenin signalling pathway, where genes such as LRP5 play essential roles in osteoblast differentiation and activity [61]. LRP5 polymorphisms have been associated with variations in spinal BMD and trabecular bone formation in childhood, illustrating the genetic basis of bone development [61]. Furthermore, interactions between CYP27B1, CYP24A1 and immune regulators such as interferons highlight the dynamic crosstalk between bone metabolism and immune signalling, where vitamin D signalling can dominate inflammatory responses to promote bone mineralization [59]. In addition to vitamin D‐related genes, the Oestrogen Receptor Alpha (ESR1) plays a pivotal role in bone remodelling, particularly in response to oestrogen, which is essential for skeletal growth, bone turnover and the prevention of postmenopausal bone loss. ESR1 encodes a nuclear receptor that, upon binding with oestrogen, activates signalling pathways influencing osteoblast proliferation and inhibiting osteoclast differentiation [62, 63].

## 6 Interaction Between Genetics and Vitamin D

### 6.1 Gene‐Vitamin D Interactions in Osteoporosis Pathogenesis

**FIGURE 2:**
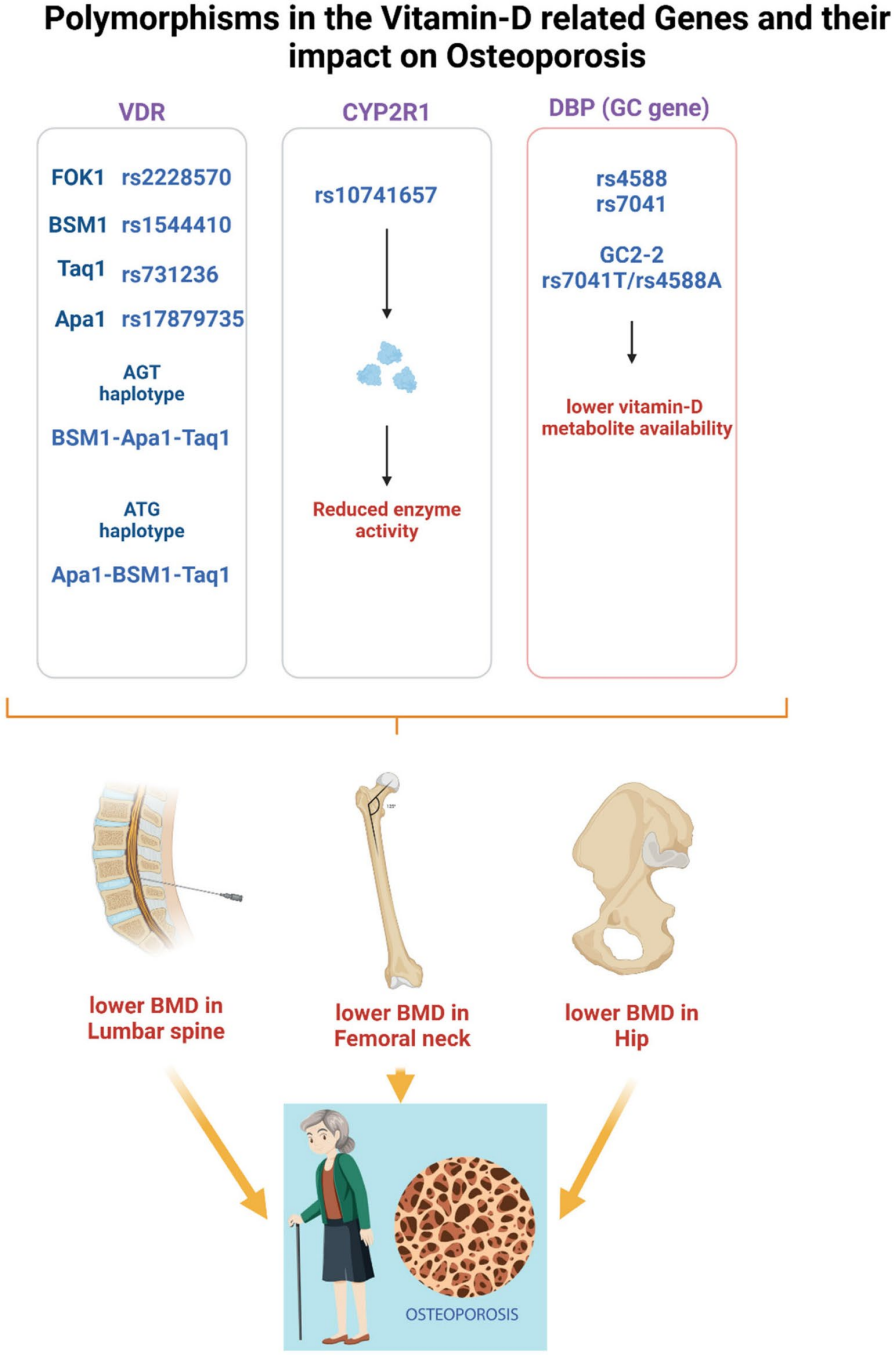
Impact of vitamin‐D‐related genes polymorphisms on osteoporosis.

Genetic polymorphisms significantly influence the metabolism, action and overall impact of vitamin D on bone health. As shown in Figure 2, these variations may occur in key components of the vitamin D pathway, including enzymes, transport proteins and receptors, ultimately affecting bone mineral density (BMD) and susceptibility to osteoporosis.

Singh et al. explored the critical role of polymorphisms in the VDR gene in determining BMD and the risk of osteoporosis, particularly in postmenopausal women. Their study highlights the impact of key VDR gene variants on BMD and their association with osteoporosis in a population of Northwest Indian women, shedding light on the genetic mechanisms underlying bone health [46]. Based on their findings, the FokI minor allele ‘T’ was significantly associated with an increased risk of osteoporosis (OR 1.60, 95% CI 1.16–2.20, *p* = 0.004), with stronger associations observed in the dominant (OR 2.32, 95% CI 1.47–3.64, *p* = 0.0006) and additive models (OR 2.41, 95% CI 1.49–3.87, *p* = 0.0006). It also showed an allele dose effect, correlating with reduced BMD at the lumbar spine (*p* = 0.009) and femoral neck (*p* = 0.036). The BsmI minor allele ‘A’ was associated with osteopenia (OR 1.53, 95% CI 1.07–2.18, *p* = 0.02) and osteoporosis (OR 1.63, 95% CI 1.19–2.23, *p* = 0.002), with the recessive model for osteoporosis yielding OR 3.15 (95% CI 1.64–6.29, *p* = 0.001) and the additive model yielding OR 3.44 (95% CI 1.67–7.08, *p* = 0.001). The TaqI minor allele ‘C’ was significantly associated with osteoporosis (OR 1.44, 95% CI 1.05–1.97, *p* = 0.02) and with reduced BMD at the lumbar spine (*p* = 0.025) and femoral neck (*p* = 0.046). While ApaI did not directly correlate with osteoporosis risk, genotype differences influenced lumbar spine BMD (*p* = 0.044). Haplotype analysis revealed the AGT (BsmI‐ApaI‐TaqI) haplotype significantly increased the risk of osteopenia (OR 2.04, 95% CI 1.03–4.08, *p* = 0.036) and osteoporosis (OR 2.90, 95% CI 1.61–5.38, *p* = 0.00005), with AGT carriers having a 2.8‐fold higher osteoporosis risk, following a recessive inheritance pattern. This haplotype also strongly correlated with reduced BMD at the lumbar spine (*p* = 0.0001) and femoral neck (*p* = 0.016). BMD measurements showed that individuals with high‐risk genotypes or haplotypes exhibited significantly lower values, such as TT genotypes for FokI (0.88 g/cm^2^ lumbar spine BMD) and CC genotypes for TaqI (0.79 g/cm^2^ femoral neck BMD). All results were adjusted for confounding factors (age, BMI, years since menopause), validated using linkage disequilibrium analyses and corrected for multiple comparisons. These findings underscore the importance of genetic screening to identify high‐risk individuals for targeted osteoporosis prevention [46]. VDR gene polymorphisms, particularly BsmI (rs1544410), ApaI (rs17879735) and TaqI (rs731236), were analysed in postmenopausal Polish women with osteoporosis to assess their association with BMD and fracture risk [64]. The study revealed that carriers of single alleles b (BsmI), a (ApaI) and T (TaqI) had significantly higher risks of non‐vertebral fractures. The B:b allele ratio in fracture cases was 1:2 compared to 1:1.5 in controls (*p* = 0.032), while the T:t ratio was 2:1 in fracture cases versus 1.5:1 in controls (*p* = 0.020). Although fractures were more common among individuals with these alleles, no direct correlation between these polymorphisms and BMD was observed. This suggests that fracture susceptibility may involve mechanisms beyond BMD, such as bone microarchitecture or remodelling efficiency. Additionally, environmental factors, including calcium intake, were highlighted as critical modulators of these genetic effects, with adequate dietary calcium reducing the impact of BsmI and TaqI polymorphisms on bone health [64]. The study by Marozik et al. investigated the relationship between five VDR gene polymorphisms (rs7975232 [ApaI], rs1544410 [BsmI], rs731236 [TaqI], rs2228570 [FokI] and rs11568820 [Cdx2]) and postmenopausal osteoporosis (PMO), bone mineral density (BMD) and serum 25‐hydroxyvitamin D (25(OH)D) levels in Belarusian women [65]. Results showed that the ApaI (A/A), BsmI (T/T) and TaqI (G/G) genotypes significantly increased PMO risk, with odds ratios (ORs) of 2.1 (95% CI 1.3–3.6, *p* < 0.05), 2.1 (95% CI 1.2–3.6, *p* < 0.01) and 2.3 (95% CI 1.4–4.0, *p* < 0.01), respectively. The A‐T‐G haplotype (ApaI, BsmI, TaqI) was strongly associated with increased PMO risk (OR 1.8, 95% CI 1.4–2.3, *p* < 0.0001), while the C‐C‐A haplotype was protective (*p* = 0.001). Individuals with these risk genotypes exhibited significantly lower lumbar spine BMD (β = −0.13 to −0.15 g/cm^2^, *p* < 0.001), while the rs11568820 A allele was protective, correlating with higher BMD (β = +0.22 g/cm^2^, *p* = 0.027). Serum 25(OH)D levels were higher in individuals with T/T (BsmI) and G/G (TaqI) genotypes (*p* < 0.01), whereas the A allele of rs11568820 was associated with lower levels (*p* = 0.027). PMO patients exhibited markedly lower lumbar spine (0.9 g/cm^2^ vs. 1.3 g/cm^2^, *p* < 0.0001) and femoral neck BMD (0.8 g/cm^2^ vs. 1.1 g/cm^2^, *p* < 0.0001) compared to controls, further underscoring the clinical significance of these genetic variants. Haplotype analysis confirmed the co‐inheritance of ApaI, BsmI and TaqI alleles as a strong predictive block for PMO and BMD variation [65]. Building on the evidence of the critical role of VDR polymorphisms in bone health, additional insights into the broader genetic regulation of vitamin D metabolism further highlight the intricate interplay between genetic variants and vitamin D activity. For example, CYP2R1 polymorphisms, including rs10741657, reduce enzyme activity and circulating 25‐hydroxyvitamin D (25(OH)D) levels, contributing to vitamin D deficiency and associated disorders such as rickets. Mutations in CYP24A1, which encodes the enzyme responsible for deactivating vitamin D metabolites, can result in hypercalcemia and nephrocalcinosis due to excess active vitamin D. Polymorphisms in the GC gene, such as rs4588 and rs7041, influence the binding and transport of vitamin D, with significant ethnic variability in their effects on bioavailability. Genome‐wide association studies (GWAS) have identified loci such as CYP24A1 (rs6013897), GC (rs2282679) and CYP2R1 (rs10741657) linked to vitamin D levels in European populations, highlighting the population‐specific nature of these associations. These genetic variations are critical in modulating the risk of disorders like osteoporosis, autoimmune diseases and cancers, emphasizing the importance of personalized medicine in addressing vitamin D deficiency and related health outcomes [66]. Moreover, it was reported that the BsmI polymorphism was associated with a 10‐year earlier decline in BMD to the fracture threshold in individuals with the BB genotype compared to bb (*p* < 0.05), while FokI was linked to a 13% lower lumbar spine BMD and greater hip bone loss in ff genotypes (4.7% vs. 0.5% for FF, *p* < 0.01). Nutritional factors further modulate these effects; calcium intake above 1000 mg/day improved BMD in Bb genotypes but had limited effects in BB genotypes, and vitamin D supplementation (10 μg/day for 2 years) produced greater BMD improvements in BB and Bb genotypes than bb (*p* < 0.01). High caffeine intake (> 300 mg/day) was associated with increased vertebral bone loss in individuals with the tt VDR genotype compared to TT. These findings highlight the complex interplay between genetic predispositions, dietary intake and environmental factors in determining osteoporosis risk, underscoring the importance of personalised nutrition and genetic screening to optimize bone health outcomes [67]. However, the findings of the Dabirnia et al. study do not support the previous research [49]. They, investigated the relationship between TaqI (rs731236) and ApaI (rs7975232) polymorphisms of the VDR gene and osteoporosis in menopausal Azari women in Zanjan province, Iran, through a case–control study involving 50 osteoporotic and 50 non‐osteoporotic women confirmed by DEXA. For ApaI, genotype frequencies were AA (48% in osteoporotic vs. 60% in controls), Aa (50% vs. 36%) and aa (2% vs. 4%), with no significant difference in genotypes (*p* = 0.37) or allele frequencies (A allele: 73% vs. 78%, *p* = 0.41). Similarly, for TaqI, genotype frequencies were TT (40% vs. 32%), Tt (48% vs. 58%) and tt (12% vs. 10%), with no significant differences in genotypes (*p* = 0.64) or allele frequencies (T allele: 64% vs. 61%, *p* = 0.66). Combined ApaI/TaqI haplotype analysis also revealed no significant association with osteoporosis (*p* = 0.563). All polymorphisms adhered to Hardy–Weinberg equilibrium and logistic regression and linkage disequilibrium analysis showed no significant relationships between these VDR polymorphisms and osteoporosis risk [49]. While this study found no associations, the authors note conflicting results in the literature, underscoring the influence of population‐specific genetic and environmental factors for future research. Another important finding was review by Bouillon and Bikle which revises key concepts in vitamin D metabolism, highlighting the regulation of CYP2R1 (25‐hydroxylase), which was previously considered constitutively expressed but is now shown to be influenced by metabolic conditions such as obesity, diabetes, and fasting [68]. In obesity, liver CYP2R1 mRNA expression and protein levels are reduced by 40% and 50%, respectively, leading to a 70% decrease in 25‐hydroxylase activity (*p* < 0.01) and ~20% lower serum 25‐hydroxyvitamin D (25OHD) levels. Similarly, diabetes reduces hepatic CYP2R1 mRNA by up to 45%, while fasting decreases it by 80% and total 25‐hydroxylase activity by 50%. These effects are mediated by the PGC1α‐ERRα pathway and epigenetic changes, including hypermethylation of CYP2R1 and CYP27B1 promoters and hypomethylation of CYP24A1, which enhances vitamin D catabolism. Polymorphisms in the GC gene encoding vitamin D‐binding protein (DBP) also affect 25OHD levels, with GC2‐2 carriers showing a 10% reduction. Clinical implications include the need for 2–3 times higher vitamin D doses in obese individuals to achieve normal 25OHD levels. These findings challenge prior dogmas, emphasizing the role of metabolic regulation and epigenetic mechanisms in vitamin D homeostasis and deficiency, particularly in conditions like obesity and diabetes [68]. In addition, vitamin D‐binding protein (VDBP) levels were found to be significantly lower in women with osteopenia and osteoporosis compared to normal controls (*p* < 0.001), with a positive correlation observed between VDBP levels and bone mineral density (BMD) (*r* = 0.23, *p* < 0.001). Receiver operating characteristic (ROC) analysis for VDBP demonstrated high diagnostic accuracy for detecting low BMD, with an AUC of 0.85 (95% CI: 0.77–0.94, *p* < 0.001), sensitivity of 70% and specificity of 75% at a cutoff of 260 μg/mL. Proteomic analysis identified VDBP as the most significantly reduced protein in osteopenic and osteoporotic samples (average fold change: −2.6, *p* < 0.001), alongside ceruloplasmin (CP) and gelsolin (GSN), which also showed lower levels in osteoporotic individuals, with AUC values of 0.69 (95% CI: 0.57–0.82, *p* = 0.003) and 0.66 (95% CI: 0.53–0.79, *p* = 0.02), respectively. Validation in a cohort of 425 postmenopausal women and an independent group of 21 women with fragility fractures confirmed VDBP's role as a non‐invasive biomarker for low BMD and fracture risk [69]. The study by Hernández‐Becerra et al. highlights the impact of GC gene polymorphisms (rs7041 and rs4588) on serum vitamin D‐binding protein (VDBP) levels and bone mineral density (BMD) [70]. Carriers of the GG genotype of rs7041 had significantly higher serum VDBP levels compared to TT carriers (284.1 μmol/L vs. 262.6 μmol/L in women, *p* < 0.001), while individuals with the A allele of rs4588 exhibited lower VDBP levels (260.0 μmol/L vs. 282.0 μmol/L, *p* < 0.001). VDBP levels positively correlated with hip BMD (*r* = 0.158, *p* < 0.001) and femoral neck BMD (*r* = 0.140, *p* < 0.001) in women, and postmenopausal women in the highest VDBP category were less likely to have low hip BMD (OR = 0.54; 95% CI: 0.33–0.77, *p* = 0.002). The GG genotype of rs7041 was associated with higher hip BMD in both premenopausal (1.058 g/cm^2^ vs. 0.998 g/cm^2^, *p* = 0.004) and postmenopausal women (0.942 g/cm^2^ vs. 0.910 g/cm^2^, *p* = 0.032), while the A allele of rs4588 correlated with lower hip BMD in premenopausal women (*p* = 0.008). Women with the GC2 haplotype (rs7041‐T/rs4588‐A) had lower femoral neck and hip BMD compared to those with GC1 haplotypes, particularly in premenopausal women. Additionally, serum VDBP levels decreased significantly with age in women (*p* < 0.001) but not in men (*p* = 0.264). These findings emphasize the influence of GC gene polymorphisms on VDBP levels and BMD, particularly in women and highlight the importance of genetic variability in bone health [70]. Also, Lauridsen et al. investigated the role of the Gc phenotype of vitamin D‐binding protein (VDBP) in fracture risk and bone mineral density (BMD) in 595 postmenopausal women from the Danish Osteoporosis Prevention Study (DOPS), finding significant variation in fracture risk among phenotypes [71]. Women with the Gc1–1 phenotype had the highest fracture risk (34%, 110/323 women, *p* = 0.017), followed by Gc1–2 (27%, 63/230 women), while Gc2–2 exhibited the lowest risk (14%, 6/42 women), with no low‐energy fractures reported in this group (*p* = 0.005). Logistic regression revealed that Gc2–2 carriers had significantly lower odds of fracture compared to Gc1–1 (OR = 0.32; 95% CI: 0.13–0.80, *p* = 0.014). Plasma Gc concentrations differed significantly among phenotypes, being highest in Gc1–1 (272 ± 2 mg/L), intermediate in Gc1–2 (249 ± 2 mg/L) and lowest in Gc2–2 (226 ± 3 mg/L, *p* < 0.001). In women with low‐energy fractures, Gc concentration negatively correlated with BMD at the lumbar spine (*r* = −0.28, *p* = 0.02), femoral neck (*r* = −0.33, *p* = 0.005) and total body (*r* = −0.34, *p* = 0.004). Although no baseline differences in BMD were observed among Gc phenotypes (*p* = 0.21–0.97), plasma alkaline phosphatase (*r* = 0.17, *p* = 0.001) and bone‐specific alkaline phosphatase (*r* = 0.12, *p* = 0.003) showed positive correlations with Gc concentration. Soluble CD163, a marker of macrophage activity, was significantly lower in Gc2–2 (2.14 ± 0.15 mg/L) compared to Gc1–1 (2.91 ± 0.19 mg/L, *p* = 0.03), suggesting reduced macrophage and osteoclast activity in Gc2–2 carriers. These findings highlight the Gc phenotype as a significant predictor of fracture risk, with Gc2–2 offering protective effects and potential for targeted prevention strategies [71]. Recently, the meta‐analysis evaluated the association of four VDR polymorphisms (FokI, BsmI, ApaI and TaqI) with osteoporosis risk across 81 studies involving 19,268 participants, revealing significant findings influenced by ethnicity and genetic models [72]. For FokI, the dominant model showed a significant association with increased osteoporosis risk (OR = 1.19, 95% CI = 1.04–1.36, *p* = 0.01, *I* ^2^ = 39.36%), indicating higher susceptibility in individuals with ‘Ff + ff’ genotypes compared to ‘FF’. While BsmI demonstrated no overall association, subgroup analysis highlighted a marginal association in Caucasians under the recessive model (OR = 1.35, 95% CI = 1.11–1.63, *p* = 0.002, *I* ^2^ = 50.07%). ApaI showed no significant associations across all genetic models (e.g., allele OR = 1.01, 95% CI = 0.87–1.17, *p* = 0.86). For TaqI, the recessive model in Caucasians showed a significant association (OR = 1.35, 95% CI = 1.11–1.63, *p* = 0.002), while no significant associations were observed in other populations or models. Ethnic variability played a key role, with Asians showing non‐significant trends for FokI and ApaI, while TaqI and BsmI were more relevant in Caucasian subgroups. High heterogeneity (*I* ^2^ > 50%) in many analyses highlighted potential differences in study design, ethnicity and environmental factors. This large‐scale meta‐analysis emphasizes the role of genetic variability in osteoporosis risk, particularly within population‐specific contexts [72]. The study conducted on 300 South Indian postmenopausal women identified significant associations between specific genetic polymorphisms and bone mineral density (BMD) [73]. The VDR BsmI GG genotype was significantly associated with lower BMD compared to AA and AG genotypes in osteoporotic women (*p* < 0.05), while the ApaI and TaqI polymorphisms showed no significant associations (*p* > 0.05). The PTH BstBI AA genotype was also linked to significantly lower BMD in osteoporotic women compared to GA and GG genotypes (*p* = 0.01), making it a potential genetic marker for osteoporosis susceptibility. No significant associations were observed for ER‐α1 PvuII polymorphism or COLIA1 Sp1 polymorphism with BMD; however, the XbaI polymorphism was significantly associated with BMD in osteopenic women (*p* = 0.03). Gene‐to‐gene interactions revealed significant associations for ApaI × BsmI and XbaI × BstBI combinations with low BMD (*p* = 0.013 and *p* = 0.008, respectively). Serum osteocalcin levels were highest in osteoporotic women (31.6 ± 9.8 ng/mL) compared to osteopenic (25.9 ± 7.5 ng/mL) and normal groups (15.1 ± 7.8 ng/mL; *p* < 0.00001). Odds ratio analysis indicated a reduced risk of osteoporosis for the BsmI AA genotype (OR = 0.3, 95% CI = 0.1–0.7, *p* = 0.01) and PvuII TT genotype (OR = 0.2, 95% CI = 0.1–0.5, *p* = 0.0009). These findings underscore the roles of VDR BsmI and PTH BstBI polymorphisms, as well as gene‐to‐gene interactions, in osteoporosis susceptibility in South Indian women, emphasising the need for population‐specific research [73].

## 7 Personalized Medicine and Vitamin D Supplementation

### 7.1 Nutrigenetics and Vitamin D Response

**FIGURE 3:**
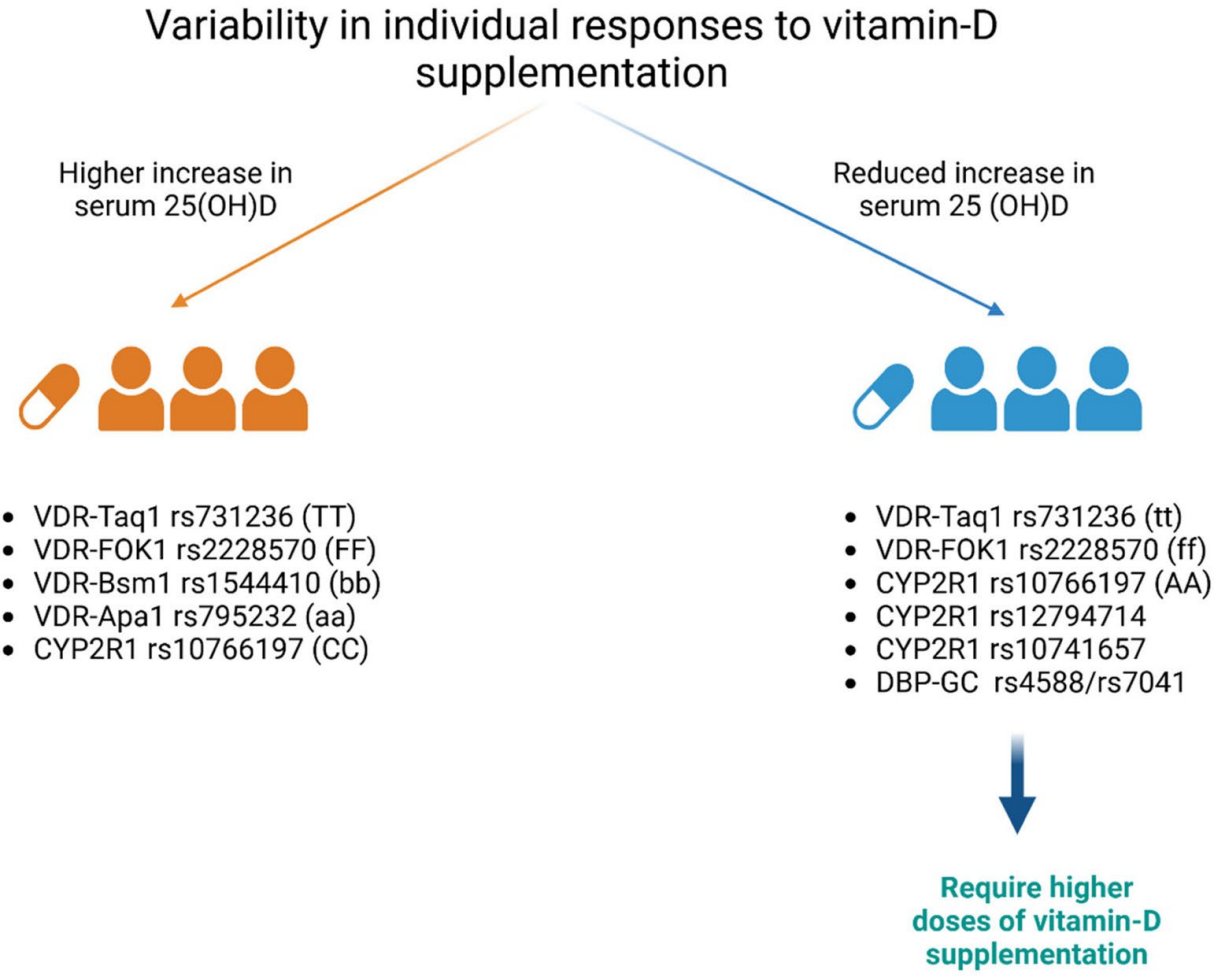
Impact of vitamin‐D‐related genes polymorphisms in individulas variablity responses to vitamin‐D supplementation.

Recent advances in the field of nutrigenetics have provided evidence on how genetic variations can impact individuals' response to dietary intakes. Polymorphisms in genes encoding proteins involved in vitamin metabolism and transport are reported to have an impact on vitamin D status after supplementation (Figure 3). For instance, a study on vitamin D supplementation response variability among 245 participants with vitamin D deficiency (25(OH)D ≤ 30 ng/mL) revealed that 11.2% were non‐responders despite personalized protocols, with mean serum 25(OH)D increasing from 13.8 ± 5.56 ng/mL to 35.74 ± 9.55 ng/mL (Δ = +21.94 ± 10.57 ng/mL) [74].

### 7.2 Genetic Contributions to Supplementation Response

Genetic factors accounted for 18.8% of variability in response, with significant contributions from DBP‐rs4588 (TT genotype: OR = 11.51, *p* < 0.001), CYP2R1‐rs10766197 (AA genotype: OR = 6.92, *p* = 0.008) and rs12794714 (AA genotype: OR = 5.09, *p* = 0.004). A composite genetic risk score (GRS) revealed that individuals with all six risk alleles had the lowest 25(OH)D increase (12.46 ± 6.1 ng/mL) compared to those with no risk alleles (24.61 ± 11.8 ng/mL, *p* = 0.041), with non‐response risk rising from OR = 1.2 for one risk allele to OR = 20.3 for six alleles. Non‐genetic factors, such as baseline 25(OH)D, physical activity and albumin, explained only 3.5% of variability, with baseline levels negatively correlating to response magnitude (*r* = −0.437, *p* < 0.001) [74].

### 7.3 SNP Influence on Supplementation Efficacy

These findings underscore the critical role of SNPs like DBP‐rs4588 and CYP2R1 variants in determining supplementation efficacy, advocating for personalized approaches to optimize vitamin D outcomes. These results reflect those of Niforou et al. who also highlight significant variability in individual responses to vitamin D supplementation, driven by genetic factors, environmental influences and baseline serum 25(OH)D levels [75]. Key genetic contributors include SNPs in the GC (vitamin D‐binding protein) gene, such as rs4588 (minor A allele associated with lower 25(OH)D levels) and rs7041 (T allele linked to reduced serum levels), with the Gc2 haplotype (AA rs4588/TT rs7041) resulting in significantly lower levels (*p* < 0.001).

### 7.4 Gene‐Specific and Ethnic Variations

Variants in DHCR7 (rs12785878, G allele), CYP2R1 (rs10741657 and rs12794714) and CYP24A1 (rs6013897) also contribute to lower 25(OH)D concentrations, with SNP rs10741657 showing a significant effect (SMD = −2.31, *p* = 3.3 × 10^−20^). Population‐specific effects were noted, such as the rs2282679 polymorphism in GC, associated with a 49% higher risk of deficiency in Caucasians (OR = 1.49, *p* < 0.0001) and the T allele of rs7041 linked to lower levels in African Americans (b = −0.93, *p* = 0.08). A genetic risk score (GRS) incorporating GC, DHCR7 and CYP2R1 SNPs revealed individuals in the highest GRS quartile had a twofold higher odds of deficiency (OR = 1.92, 95% CI = 1.70–2.16) [75].

### 7.5 Vitamin D Receptor (VDR) Polymorphisms

Significant variability in individual responses to vitamin D supplementation, driven by genetic and non‐genetic factors, including baseline serum 25(OH)D levels, BMI, health status and SNPs in VDR genes, was also shown. For instance, FokI (rs2228570) was associated with a higher increase in serum 25(OH)D levels for the ff genotype in younger individuals (< 55 years, WMD: 10.53 ng/mL, 95% CI: 7.72–13.34, *p* = 0.006) and those with BMI ≥ 30 (WMD: 16.51 ng/mL, 95% CI: 13.05–19.98, *p* = 0.001). Similarly, the BsmI (rs1544410) bb genotype showed greater elevation compared to BB + Bb genotypes (WMD: 7.59 ng/mL, 95% CI: 6.22–8.96, *p* < 0.001), particularly in older adults (≥ 55 years) and individuals with higher BMI. TaqI (rs731236) and ApaI (rs7975232) also exhibited genotype‐specific responses, with the tt and aa genotypes showing reduced and increased efficacy, respectively, particularly in younger, obese and Asian populations (ApaI WMD: 20.6 ng/mL, 95% CI: 17.51–23.7, *p* < 0.001).

### 7.6 Clinical Studies and Meta‐Analyses

Doses of 30,000–50,000 IU/week significantly improved serum 25(OH)D levels in certain genotypes, whereas doses exceeding 50,000 IU/week showed diminishing returns. Population‐specific differences, particularly between Asian and non‐Asian groups, emphasize the need for tailored supplementation strategies [76]. Interestingly, a study on breast cancer survivors (198 participants) receiving 4,000 IU/day of vitamin D3 for 12 weeks revealed significant variability in plasma 25(OH)D responses, influenced by genetic variations in VDR SNPs (FokI, BsmI, TaqI, ApaI and Cdx2) [77].

### 7.7 Genotype‐Specific Outcomes

Participants with the FokI ff genotype exhibited smaller increases in 25(OH)D. In contrast, those with the FF genotype showed greater improvements following supplementation. For TaqI, the tt genotype was linked to lower plasma levels after supplementation, whereas the ApaI aa genotype demonstrated the most pronounced response. Similarly, the Cdx2 GG genotype showed stronger responses compared to AG or AA genotypes [77]. This also accords with a study that showed that individuals with the BsmI bb genotype showed better serum 25(OH)D levels post‐supplementation, while those with the FokI ff genotype exhibited reduced responses compared to FF genotype carriers.

### 7.8 Genetic Risk Scoring and Population Differences

Asian populations demonstrated distinct supplementation outcomes, influenced by the prevalence of specific genotypes like TaqI, and higher dosages (e.g., > 4000 IU/day) were required to improve responses in genetic risk carriers. A Genetic Risk Score (GRS) combining multiple SNPs was more effective in predicting supplementation efficacy, with higher GRS scores correlating with reduced responses to standard doses [78]. Moreover, significant variability in individual responses to vitamin D supplementation was influenced by SNPs in the GC gene (e.g., rs4588 and rs7041) and other related genes [79].

### 7.9 Impact of GC and CYP2R1 Variants

Individuals carrying risk alleles, such as the rs4588 AA or rs7041 TT genotypes, exhibited reduced increases in serum 25(OH)D levels post‐supplementation compared to non‐risk carriers, with AA genotype carriers showing a mean increase of 18.5 ng/mL (95% CI: 15.3–21.7) versus 25.2 ng/mL (95% CI: 22.1–28.3) in CC carriers (*p* < 0.05). A Genetic Risk Score (GRS) incorporating variants from GC, CYP2R1 and VDR genes improved the predictability of serum vitamin D changes by 35% compared to non‐genetic models. Subjects with higher GRS required higher supplementation doses (≥ 4000 IU/day) to achieve optimal serum levels, as standard doses (≤ 2000 IU/day) were insufficient. Population‐specific differences in SNP prevalence were noted, with Asian and European cohorts exhibiting distinct allele frequencies and responses to supplementation.

### 7.10 Arab Population Study and Meta‐Analysis

In addition, a study on 100 healthy Arab women receiving 50,000 IU of vitamin D3 weekly for 12 weeks revealed significant variability in serum 25(OH)D responses, influenced by genetic polymorphisms [12]. VDR rs731236 (TaqI) was significantly associated with vitamin D sufficiency, with GG genotype carriers showing the largest serum 25(OH)D increase and a higher likelihood of achieving sufficiency compared to AG and AA genotypes (chi‐square = 8.868, *p* = 0.0119). Similarly, CYP2R1 rs7116978 was linked to supplementation efficacy, as CC genotype carriers were more likely to achieve sufficiency than TT or CT genotypes (chi‐square = 7.272, *p* = 0.0264 for deficient vs. sufficient). Functional analysis suggested that rs7116978 alters transcription factor binding sites, potentially modifying vitamin D metabolism via interactions with TCF4 and the glucocorticoid receptor. Regional differences were noted, as the favourable G allele for rs731236 was less frequent in the cohort compared to global populations [12].

### 7.11 Meta‐Analysis on VDR Effects and Bone Response

Recently, a meta‐analysis evaluating the impact of VDR gene polymorphisms (BsmI, TaqI, ApaI and FokI) on individual responses to vitamin D supplementation revealed that the TaqI polymorphism significantly influenced outcomes, with individuals carrying the variant allele (Tt + tt genotype) demonstrating better responses than TT genotype carriers (*p* = 0.02) [80]. Similarly, the FokI FF genotype was associated with a significantly better response compared to variant allele carriers (Ff + ff; *p* < 0.001). In contrast, BsmI and ApaI polymorphisms showed no significant associations with supplementation response (*p* = 0.81 and *p* = 0.63, respectively). The analysis included 1038 participants across eight studies with an average follow‐up of 7.4 months, spanning diverse age ranges (10–78 years).

### 7.12 Bone Density Outcomes and Pharmacogenomics

Importantly, the study demonstrates that VDR polymorphisms significantly influence skeletal responses to vitamin D supplementation, with implications for personalized osteoporosis prevention and treatment strategies [81]. The BsmI polymorphism showed the largest increases in bone mineral content (BMC) and bone mineral density (BMD) in individuals with the bb genotype, including a 16.4% ± 10.7% increase in lumbar spine BMC compared to 8.0% ± 7.4% in the BB genotype (*p* = 0.02) and a 6.9% ± 8.5% increase in femoral neck BMC versus 1.4% ± 5.7% (*p* = 0.04). Similarly, the TaqI TT genotype demonstrated significantly higher responses, with lumbar spine BMC increasing by 16.5% ± 10.4% compared to 7.8% ± 6.9% in the tt genotype (*p* = 0.02) and total body BMD rising by 3.8% ± 3.8% versus 1.1% ± 3.2% (*p* = 0.03). The ApaI polymorphism had no significant effect on BMC or BMD. Notably, skeletal responses were genotype‐dependent and independent of serum 25(OH)D levels, suggesting direct genetic modulation.

Adolescents with BB and tt genotypes exhibited suboptimal responses, highlighting the need for tailored supplementation protocols to optimize bone mass accrual [81]. These findings underscore the importance of integrating VDR genotyping into personalized supplementation and treatment strategies to prevent osteoporosis, particularly in genetically predisposed individuals. Also, the significant impact of genetic factors, particularly variations in the VDR gene (e.g., rs2228570 [FokI] and rs1544410 [BsmI]), on individual responses to vitamin D supplementation and osteoporosis treatment, with genotypes like FokI FF and BsmI bb associated with superior bone mineral density (BMD) outcomes was highlighted [82]. Pharmacogenomics further reveals that variations in drug‐metabolising enzymes (e.g., CYP2C9, CYP3A4) and drug transporters influence the efficacy of osteoporosis medications such as bisphosphonates and SERMs, necessitating dose adjustments or alternative treatments for patients with unfavourable genotypes. Polygenic risk scores combining markers like VDR, COLIA1 and LRP5 enhance risk prediction for osteoporosis and fractures, enabling targeted interventions for high‐risk individuals. Tailored strategies, including higher vitamin D doses for those with absorption‐related genetic predispositions and personalized exercise and dietary plans, optimize bone health outcomes. Despite challenges such as the high cost of genetic testing and ethical concerns regarding data privacy, advancements in genomics and collaborative efforts hold promise for integrating personalized medicine into osteoporosis prevention and treatment, revolutionizing care for at‐risk populations [82].

## 8 Clinical Implications and Future Perspectives

### 8.1 Implications for Public Health

Addressing vitamin D deficiency on a population level requires a multifaceted approach integrating supplementation, food fortification, public health campaigns and targeted interventions. Supplementation remains a cornerstone strategy, particularly for high‐risk groups such as the elderly, pregnant women and individuals with limited sun exposure, with daily intakes of 800–2000 IU proving effective in maintaining optimal serum levels [83, 84]. Combined supplementation of vitamin D and calcium has been shown to reduce hip and non‐vertebral fractures by 16% and 14%, respectively, among older adults. Mandatory food fortification policies, particularly for staple foods like dairy products, edible oils and cereals, have significantly improved vitamin D status in countries adopting these measures [83]. For low‐ and middle‐income countries, fortified foods serve as an essential strategy where natural dietary sources are scarce. Public health campaigns promoting safe sun exposure and the importance of dietary and supplemental vitamin D further enhance awareness and adherence. Targeted interventions, such as incorporating vitamin D supplementation into vaccination programmes or addressing deficiency in regions with > 20% prevalence of serum 25(OH)D levels below 30 nmol/L, ensure improved outreach and adherence in vulnerable populations [84].

Screening for genetic risk factors enhances the precision of vitamin D deficiency management, particularly in high‐risk groups. Genetic testing for polymorphisms in vitamin D metabolism and receptor genes, such as VDR (FokI, BsmI, TaqI), allows for the identification of individuals with reduced absorption or metabolic efficiency, necessitating higher supplementation doses [83, 84]. The use of polygenic risk scores (PRS) that incorporate markers from genes like GC, CYP2R1 and VDR has proven effective in predicting deficiency and tailoring interventions. High PRS scores have been associated with a greater need for personalized supplementation and treatment protocols [83]. Regional variability in allele frequencies highlights the importance of population‐specific screening and interventions, with strategies tailored to the genetic risk profiles of diverse groups, such as a higher prevalence of risk alleles in Asian and African populations. Integrating genetic screening into clinical practice enables tailored supplementation and pharmacological treatments, optimizing vitamin D status and preventing osteoporosis, particularly in individuals genetically predisposed to poor bone health outcomes [23].

### 8.2 Integration of Genomics Into Clinical Practice

Genetic testing plays a pivotal role in the management of osteoporosis by enabling precision in diagnosis, risk stratification and treatment personalization. Monogenic disorders such as osteogenesis imperfecta and hypophosphatasia, caused by mutations in COL1A1, COL1A2 and ALPL, can present as osteoporosis in adults, underscoring the utility of genetic diagnostics in distinguishing these conditions. GWAS have identified over 500 loci, including key variants in LRP5 and WNT1, associated with bone mineral density (BMD), explaining approximately 25% of its variance [85, 86]. Polygenic risk scores (PRS) that combine multiple genetic markers, such as VDR and LRP5, enable stratification of individuals by fracture risk, facilitating early and targeted intervention strategies [87]. PRS enhance fracture risk prediction by integrating the small additive effects of many SNPs into a cumulative score, which captures more genetic variability than any single marker and improves risk stratification when combined with clinical factors like age, sex and prior fractures. However, current limitations include the modest variance explained by PRS, lack of transferability across ethnic groups due to population‐specific allele frequencies, and the need for standardization of PRS construction and validation. Further research is necessary to improve predictive accuracy and clinical utility, especially in underrepresented populations. Furthermore, next‐generation sequencing (NGS) has improved the detection of rare disease‐causing variants (DCVs), allowing tailored therapies for familial osteoporosis and atypical fractures [88]. The integration of genetic testing into clinical practice has thus enhanced the ability to predict, prevent and manage osteoporosis in diverse populations.

However, the implementation of genetic testing in osteoporosis care faces ethical considerations and accessibility challenges. High costs limit access for underserved populations, creating disparities in the availability of advanced diagnostics, which could be mitigated by universal coverage policies and subsidies [86]. Privacy and data security concerns surrounding the collection and storage of genetic information necessitate robust protections and adherence to ethical guidelines [85]. Additionally, informed consent and access to comprehensive genetic counselling remain critical, as patients require guidance to understand test results and their implications for family members [85, 86]. Cultural and societal barriers, including fears of stigmatization and discrimination, further hinder widespread adoption, emphasizing the need for awareness campaigns and community engagement. Standardized guidelines for the integration of genetic testing into clinical practice are essential to avoid over‐reliance on genetic data without considering the clinical context [88]. Addressing these challenges will ensure equitable and ethical use of genetic testing to optimize osteoporosis prevention and treatment strategies.

### 8.3 Future Research Priorities

Large‐scale studies investigating gene‐vitamin D interactions have provided critical insights into the genetic determinants of vitamin D metabolism and their implications for health outcomes. Genome‐wide association studies (GWAS) have identified multiple loci associated with serum 25(OH)D concentrations, including genes involved in vitamin D transport (GC), hydroxylation (CYP2R1, CYP24A1) and receptor activity (VDR). Variants such as GC rs4588 and rs7041 have been shown to significantly influence circulating 25(OH)D levels, with carriers of risk alleles exhibiting reduced serum levels even after supplementation. Similarly, polymorphisms in CYP2R1, such as rs10741657, are associated with differences in vitamin D hydroxylation efficiency, further contributing to variability in serum levels [66]. These findings underscore the importance of gene–environment interactions, where genetic predisposition modulates the impact of factors such as supplementation, diet and sun exposure on vitamin D status. Population‐specific studies have also revealed regional differences in allele frequencies, emphasizing the need for geographically tailored strategies to address vitamin D deficiency. Such large‐scale genetic analyses provide a foundation for integrating genetic risk scores (GRS) into personalized interventions for optimizing vitamin D status and reducing the risk of related diseases.

Interventional studies incorporating genetic stratification have demonstrated the value of precision approaches in optimizing vitamin D supplementation and osteoporosis management. By stratifying participants based on genetic profiles, such as VDR (FokI, BsmI, TaqI) and GC polymorphisms, these studies have revealed significant genotype‐dependent responses to supplementation. For instance, carriers of the VDR FokI ff genotype or GC rs4588 AA genotype exhibit lower increases in serum 25(OH)D levels following supplementation compared to those with non‐risk genotypes, necessitating higher doses to achieve sufficiency [77]. Tailored interventions based on genetic stratification have been shown to enhance BMD outcomes, particularly in high‐risk groups with genetic predispositions to low vitamin D metabolism or poor skeletal response. Moreover, PRS integrating multiple loci have been used to identify individuals at greater risk of vitamin D deficiency or fractures, guiding more aggressive prevention strategies. While these findings highlight the potential of genetic stratification in improving clinical outcomes, challenges such as the high cost of genetic testing, limited accessibility and the need for standardized protocols remain significant barriers to widespread implementation. Future interventional studies should aim to address these challenges by leveraging advanced sequencing technologies, improving cost efficiency and incorporating diverse populations to refine and validate genetic‐based approaches to vitamin D and osteoporosis management.

## 9 Conclusion

Osteoporosis is a multifactorial disease influenced by the intricate interplay of genetics and vitamin D. Understanding this interaction offers opportunities for advancing personalized medicine approaches, optimizing supplementation strategies and mitigating osteoporosis risk in vulnerable populations. Continued research on gene–environment interactions and emerging genomic tools may contribute significantly to more precise and effective osteoporosis prevention strategies. Moreover, integrating genetic screening into routine clinical practice can enable earlier identification of at‐risk individuals, paving the way for tailored interventions. Collaborative efforts across genomics, nutrition and public health will be pivotal in addressing the growing global burden of osteoporosis.

## Author Contributions

**Sepideh Abdollahi:** project administration (equal). **Forough Taheri:** supervision (equal). **Amirhossein Sangi Nasab Lahijan:** validation (equal). **Saba Hatefi Shoga:** data curation (equal). **Ali Didehban:** writing – review and editing (equal). **Saeid Doaei:** conceptualization (equal), supervision (equal).

## Ethics Statement

The authors have nothing to report.

## Conflicts of Interest

The authors declare no conflicts of interest.

## Data Availability

Datasets used and/or analysed during the current study are available from the corresponding author on reasonable requests.
